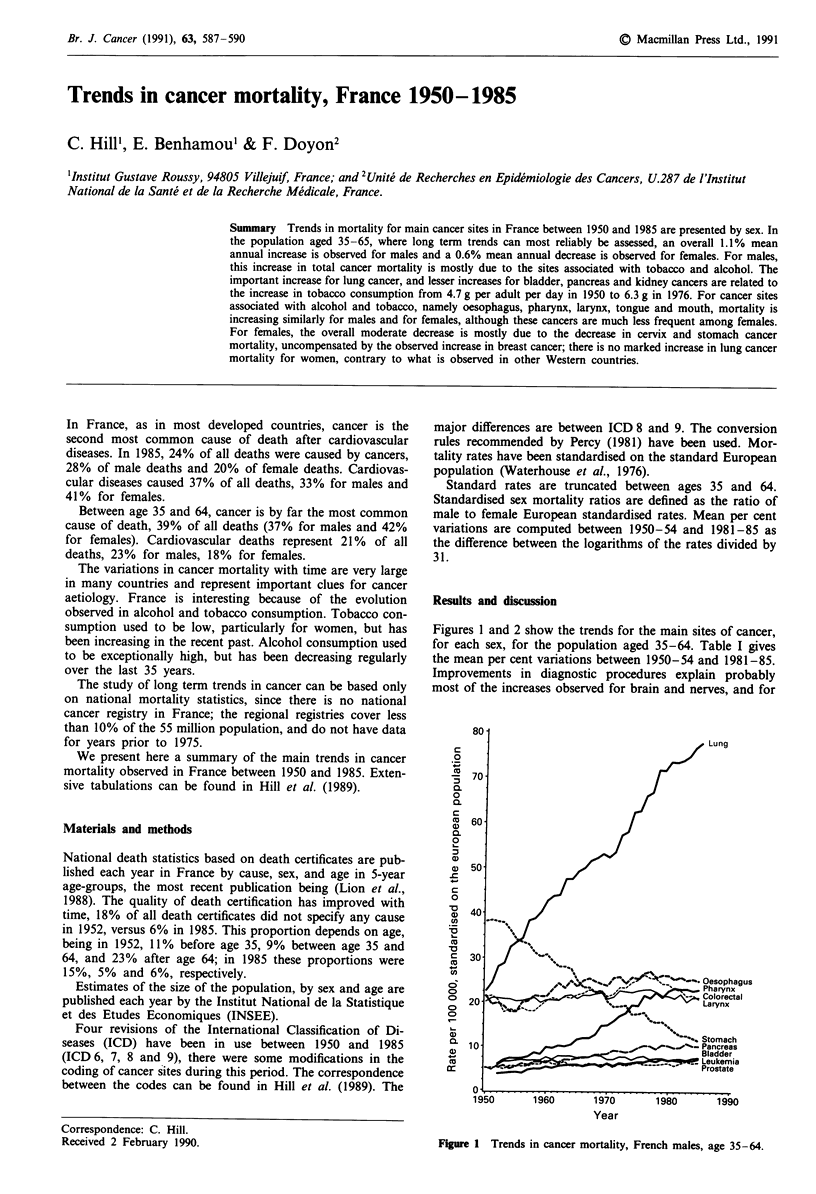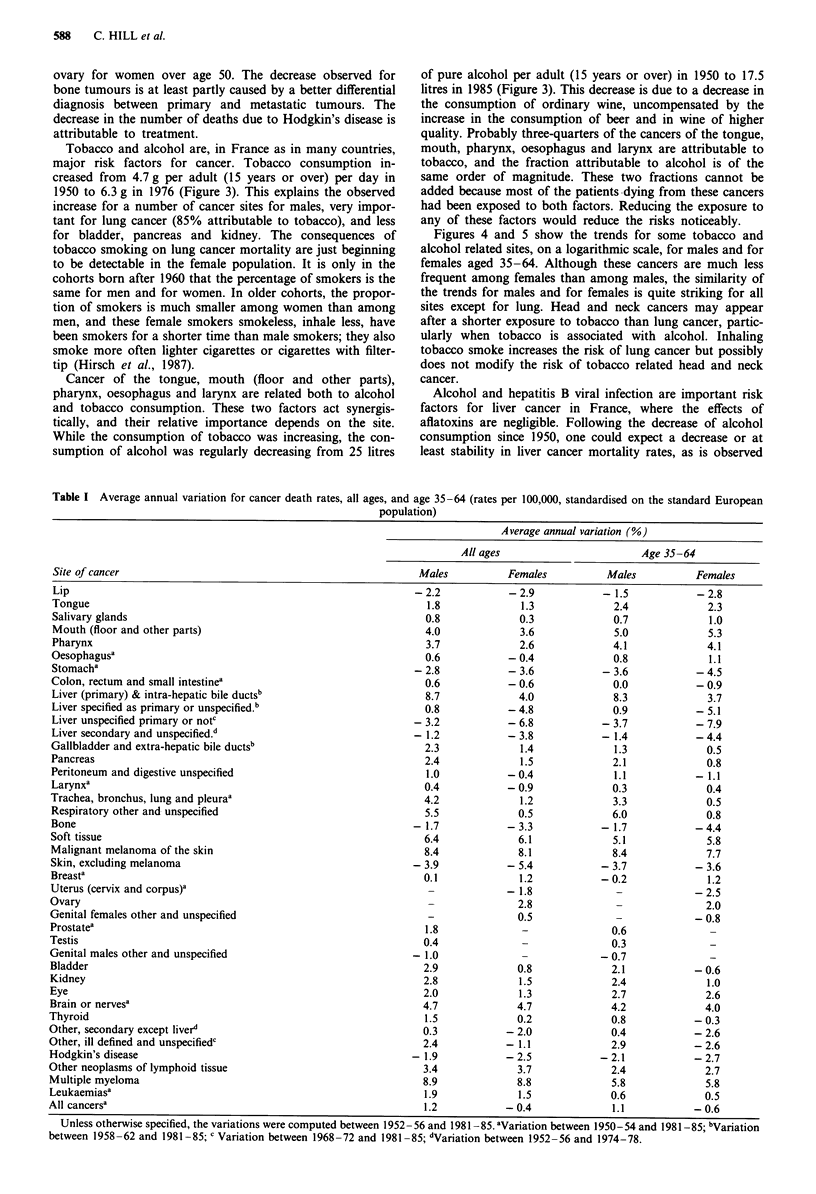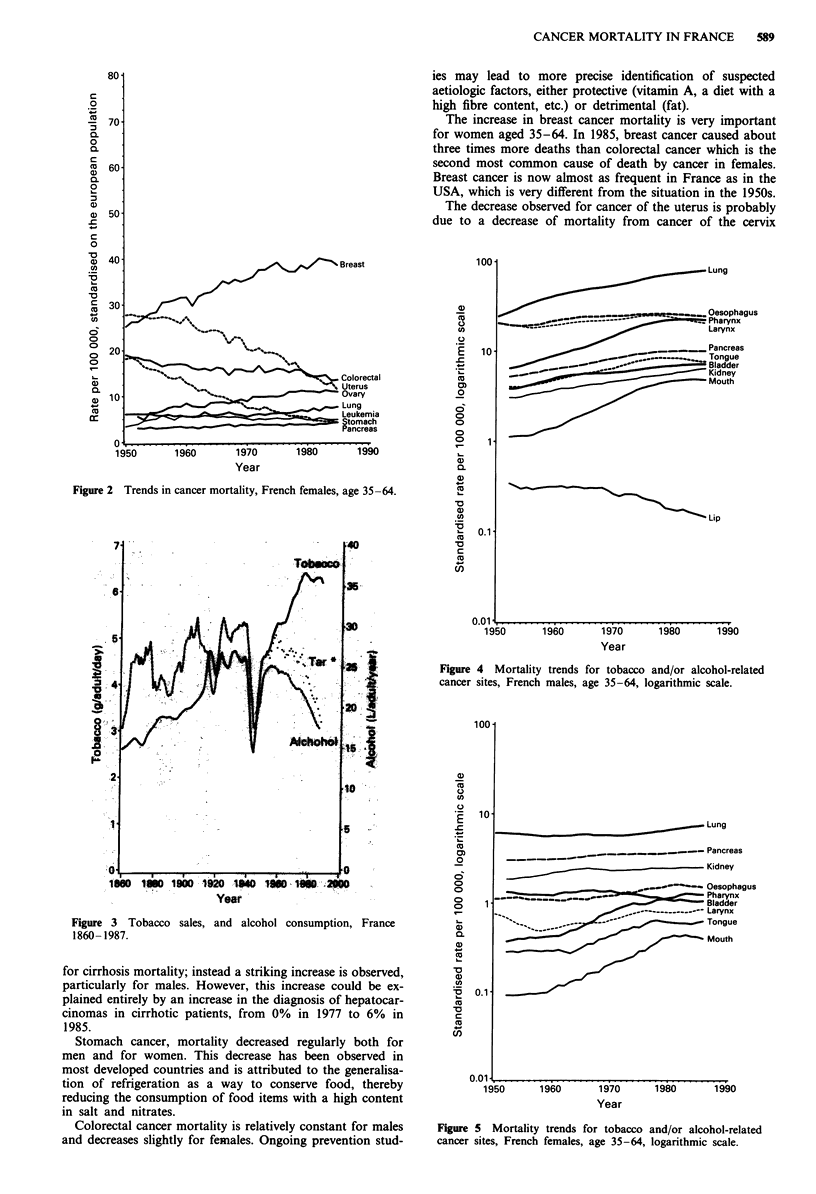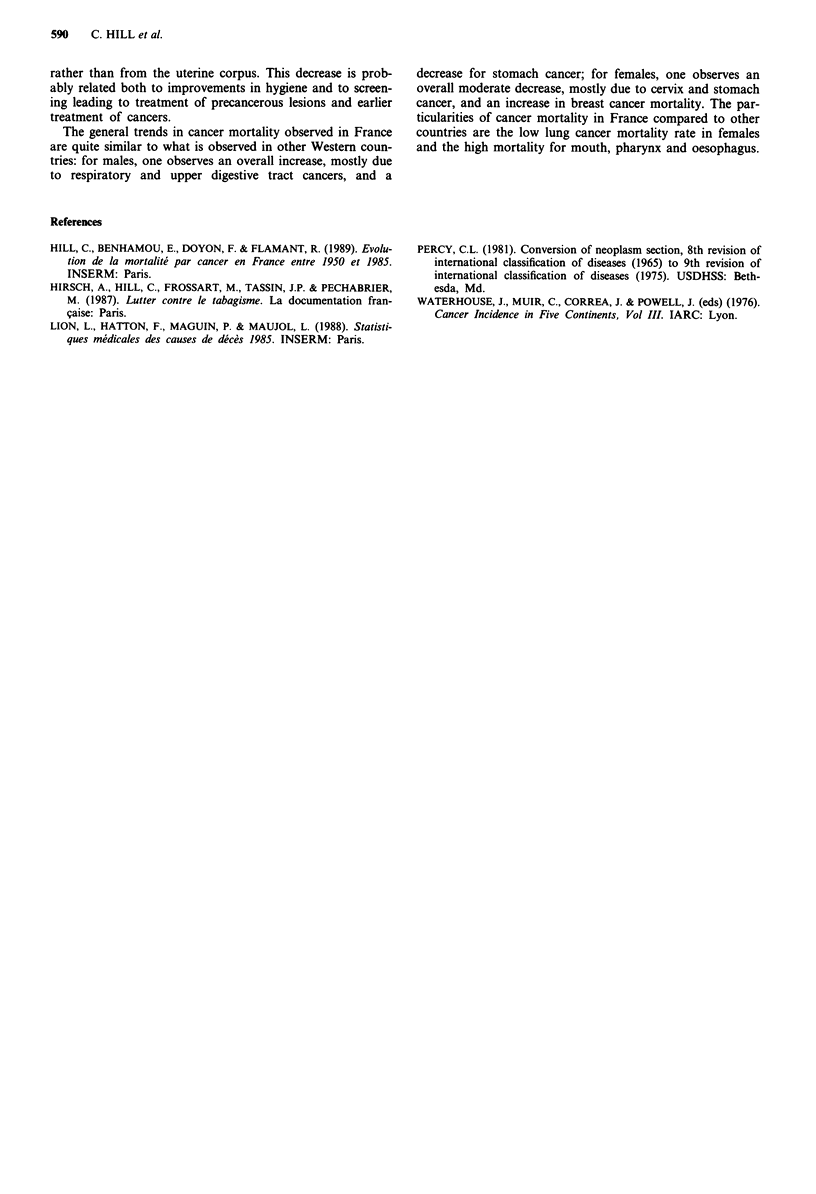# Trends in cancer mortality, France 1950-1985.

**DOI:** 10.1038/bjc.1991.136

**Published:** 1991-04

**Authors:** C. Hill, E. Benhamou, F. Doyon

**Affiliations:** Institut Gustave Roussy, Villejuif, France.

## Abstract

Trends in mortality for main cancer sites in France between 1950 and 1985 are presented by sex. In the population aged 35-65, where long term trends can most reliably be assessed, an overall 1.1% mean annual increase is observed for males and a 0.6% mean annual decrease is observed for females. For males, this increase in total cancer mortality is mostly due to the sites associated with tobacco and alcohol. The important increase for lung cancer, and lesser increases for bladder, pancreas and kidney cancers are related to the increase in tobacco consumption from 4.7 g per adult per day in 1950 to 6.3 g in 1976. For cancer sites associated with alcohol and tobacco, namely oesophagus, pharynx, larynx, tongue and mouth, mortality is increasing similarly for males and for females, although these cancers are much less frequent among females. For females, the overall moderate decrease is mostly due to the decrease in cervix and stomach cancer mortality, uncompensated by the observed increase in breast cancer; there is no marked increase in lung cancer mortality for women, contrary to what is observed in other Western countries.


Br. J. Cancer (1991), 63, 587-590 © Macmillan Press Ltd., 1991

### Trends in cancer mortality, France 1950-1985

C. Hill[1], E. Benhamou[1] & F. Doyon[2]

[1]*Institut Gustave Roussy, 94805 Villejuif, France; and* [2]*Unité de Recherches en Epidémiologie des Cancers, U.287 de l'Institut National de la Santé et de la Recherche Médicale, France.*

**Summary** Trends in mortality for main cancer sites in France between 1950 and 1985 are presented by sex. In the population aged 35-65, where long term trends can most reliably be assessed, an overall 1.1% mean annual increase is observed for males and a 0.6% mean annual decrease is observed for females. For males, this increase in total cancer mortality is mostly due to the sites associated with tobacco and alcohol. The important increase for lung cancer, and lesser increases for bladder, pancreas and kidney cancers are related to the increase in tobacco consumption from 4.7 g per adult per day in 1950 to 6.3 g in 1976. For cancer sites associated with alcohol and tobacco, namely oesophagus, pharynx, larynx, tongue and mouth, mortality is increasing similarly for males and for females, although these cancers are much less frequent among females. For females, the overall moderate decrease is mostly due to the decrease in cervix and stomach cancer mortality, uncompensated by the observed increase in breast cancer; there is no marked increase in lung cancer mortality for women, contrary to what is observed in other Western countries.

In France, as in most developed countries, cancer is the second most common cause of death after cardiovascular diseases. In 1985, 24% of all deaths were caused by cancers, 28% of male deaths and 20% of female deaths. Cardiovascular diseases caused 37% of all deaths, 33% for males and 41% for females.

Between age 35 and 64, cancer is by far the most common cause of death, 39% of all deaths (37% for males and 42% for females). Cardiovascular deaths represent 21% of all deaths, 23% for males, 18% for females.

The variations in cancer mortality with time are very large in many countries and represent important clues for cancer aetiology. France is interesting because of the evolution observed in alcohol and tobacco consumption. Tobacco consumption used to be low, particularly for women, but has been increasing in the recent past. Alcohol consumption used to be exceptionally high, but has been decreasing regularly over the last 35 years.

The study of long term trends in cancer can be based only on national mortality statistics, since there is no national cancer registry in France; the regional registries cover less than 10% of the 55 million population, and do not have data for years prior to 1975.

We present here a summary of the main trends in cancer mortality observed in France between 1950 and 1985. Extensive tabulations can be found in Hill *et al.* (1989).

### Materials and methods

National death statistics based on death certificates are published each year in France by cause, sex, and age in 5-year age-groups, the most recent publication being (Lion *et al.*, 1988). The quality of death certification has improved with time, 18% of all death certificates did not specify any cause in 1952, versus 6% in 1985. This proportion depends on age, being in 1952, 11% before age 35, 9% between age 35 and 64, and 23% after age 64; in 1985 these proportions were 15%, 5% and 6%, respectively.

Estimates of the size of the population, by sex and age are published each year by the Institut National de la Statistique et des Etudes Economiques (INSEE).

Four revisions of the International Classification of Diseases (ICD) have been in use between 1950 and 1985 (ICD 6, 7, 8 and 9), there were some modifications in the coding of cancer sites during this period. The correspondence between the codes can be found in Hill *et al.* (1989). The major differences are between ICD 8 and 9. The conversion rules recommended by Percy (1981) have been used. Mortality rates have been standardised on the standard European population (Waterhouse *et al.*, 1976).

Standard rates are truncated between ages 35 and 64. Standardised sex mortality ratios are defined as the ratio of male to female European standardised rates. Mean per cent variations are computed between 1950-54 and 1981-85 as the difference between the logarithms of the rates divided by 31.

### Results and discussion

Figures 1 and 2 show the trends for the main sites of cancer, for each sex, for the population aged 35-64. Table I gives the mean per cent variations between 1950-54 and 1981-85. Improvements in diagnostic procedures explain probably most of the increases observed for brain and nerves, and for

**Figure 1** Trends in cancer mortality, French males, age 35-64.

Correspondence: C. Hill.
Received 2 February 1990.

ovary for women over age 50. The decrease observed for bone tumours is at least partly caused by a better differential diagnosis between primary and metastatic tumours. The decrease in the number of deaths due to Hodgkin's disease is attributable to treatment.

Tobacco and alcohol are, in France as in many countries, major risk factors for cancer. Tobacco consumption increased from 4.7 g per adult (15 years or over) per day in 1950 to 6.3 g in 1976 (Figure 3). This explains the observed increase for a number of cancer sites for males, very important for lung cancer (85% attributable to tobacco), and less for bladder, pancreas and kidney. The consequences of tobacco smoking on lung cancer mortality are just beginning to be detectable in the female population. It is only in the cohorts born after 1960 that the percentage of smokers is the same for men and for women. In older cohorts, the proportion of smokers is much smaller among women than among men, and these female smokers smokeless, inhale less, have been smokers for a shorter time than male smokers; they also smoke more often lighter cigarettes or cigarettes with filter-tip (Hirsch et al., 1987).

Cancer of the tongue, mouth (floor and other parts), pharynx, oesophagus and larynx are related both to alcohol and tobacco consumption. These two factors act synergistically, and their relative importance depends on the site. While the consumption of tobacco was increasing, the consumption of alcohol was regularly decreasing from 25 litres of pure alcohol per adult (15 years or over) in 1950 to 17.5 litres in 1985 (Figure 3). This decrease is due to a decrease in the consumption of ordinary wine, uncompensated by the increase in the consumption of beer and in wine of higher quality. Probably three-quarters of the cancers of the tongue, mouth, pharynx, oesophagus and larynx are attributable to tobacco, and the fraction attributable to alcohol is of the same order of magnitude. These two fractions cannot be added because most of the patients dying from these cancers had been exposed to both factors. Reducing the exposure to any of these factors would reduce the risks noticeably.

Figures 4 and 5 show the trends for some tobacco and alcohol related sites, on a logarithmic scale, for males and for females aged 35–64. Although these cancers are much less frequent among females than among males, the similarity of the trends for males and for females is quite striking for all sites except for lung. Head and neck cancers may appear after a shorter exposure to tobacco than lung cancer, particularly when tobacco is associated with alcohol. Inhaling tobacco smoke increases the risk of lung cancer but possibly does not modify the risk of tobacco related head and neck cancer.

Alcohol and hepatitis B viral infection are important risk factors for liver cancer in France, where the effects of aflatoxins are negligible. Following the decrease of alcohol consumption since 1950, one could expect a decrease or at least stability in liver cancer mortality rates, as is observed

**Table I** Average annual variation for cancer death rates, all ages, and age 35–64 (rates per 100,000, standardised on the standard European population)

| | *Average annual variation (%)* | | | |
|---|---|---|---|---|
| | *All ages* | | *Age 35–64* | |
| *Site of cancer* | *Males* | *Females* | *Males* | *Females* |
| Lip | – 2.2 | – 2.9 | – 1.5 | – 2.8 |
| Tongue | 1.8 | 1.3 | 2.4 | 2.3 |
| Salivary glands | 0.8 | 0.3 | 0.7 | 1.0 |
| Mouth (floor and other parts) | 4.0 | 3.6 | 5.0 | 5.3 |
| Pharynx | 3.7 | 2.6 | 4.1 | 4.1 |
| Oesophagus[a] | 0.6 | – 0.4 | 0.8 | 1.1 |
| Stomach[a] | – 2.8 | – 3.6 | – 3.6 | – 4.5 |
| Colon, rectum and small intestine[a] | 0.6 | – 0.6 | 0.0 | – 0.9 |
| Liver (primary) & intra-hepatic bile ducts[b] | 8.7 | 4.0 | 8.3 | 3.7 |
| Liver specified as primary or unspecified.[b] | 0.8 | – 4.8 | 0.9 | – 5.1 |
| Liver unspecified primary or not[c] | – 3.2 | – 6.8 | – 3.7 | – 7.9 |
| Liver secondary and unspecified.[d] | – 1.2 | – 3.8 | – 1.4 | – 4.4 |
| Gallbladder and extra-hepatic bile ducts[b] | 2.3 | 1.4 | 1.3 | 0.5 |
| Pancreas | 2.4 | 1.5 | 2.1 | 0.8 |
| Peritoneum and digestive unspecified | 1.0 | – 0.4 | 1.1 | – 1.1 |
| Larynx[a] | 0.4 | – 0.9 | 0.3 | 0.4 |
| Trachea, bronchus, lung and pleura[a] | 4.2 | 1.2 | 3.3 | 0.5 |
| Respiratory other and unspecified | 5.5 | 0.5 | 6.0 | 0.8 |
| Bone | – 1.7 | – 3.3 | – 1.7 | – 4.4 |
| Soft tissue | 6.4 | 6.1 | 5.1 | 5.8 |
| Malignant melanoma of the skin | 8.4 | 8.1 | 8.4 | 7.7 |
| Skin, excluding melanoma | – 3.9 | – 5.4 | – 3.7 | – 3.6 |
| Breast[a] | 0.1 | 1.2 | – 0.2 | 1.2 |
| Uterus (cervix and corpus)[a] | – | – 1.8 | – | – 2.5 |
| Ovary | – | 2.8 | – | 2.0 |
| Genital females other and unspecified | – | 0.5 | – | – 0.8 |
| Prostate[a] | 1.8 | – | 0.6 | – |
| Testis | 0.4 | – | 0.3 | – |
| Genital males other and unspecified | – 1.0 | – | – 0.7 | – |
| Bladder | 2.9 | 0.8 | 2.1 | – 0.6 |
| Kidney | 2.8 | 1.5 | 2.4 | 1.0 |
| Eye | 2.0 | 1.3 | 2.7 | 2.6 |
| Brain or nerves[a] | 4.7 | 4.7 | 4.2 | 4.0 |
| Thyroid | 1.5 | 0.2 | 0.8 | – 0.3 |
| Other, secondary except liver[d] | 0.3 | – 2.0 | 0.4 | – 2.6 |
| Other, ill defined and unspecified[c] | 2.4 | – 1.1 | 2.9 | – 2.6 |
| Hodgkin's disease | – 1.9 | – 2.5 | – 2.1 | – 2.7 |
| Other neoplasms of lymphoid tissue | 3.4 | 3.7 | 2.4 | 2.7 |
| Multiple myeloma | 8.9 | 8.8 | 5.8 | 5.8 |
| Leukaemias[a] | 1.9 | 1.5 | 0.6 | 0.5 |
| All cancers[a] | 1.2 | – 0.4 | 1.1 | – 0.6 |

Unless otherwise specified, the variations were computed between 1952–56 and 1981–85. [a]Variation between 1950–54 and 1981–85; [b]Variation between 1958–62 and 1981–85; [c] Variation between 1968–72 and 1981–85; [d]Variation between 1952–56 and 1974–78.

**Figure 2** Trends in cancer mortality, French females, age 35-64.

**Figure 3** Tobacco sales, and alcohol consumption, France 1860-1987.

for cirrhosis mortality; instead a striking increase is observed, particularly for males. However, this increase could be explained entirely by an increase in the diagnosis of hepatocarcinomas in cirrhotic patients, from 0% in 1977 to 6% in 1985.

Stomach cancer, mortality decreased regularly both for men and for women. This decrease has been observed in most developed countries and is attributed to the generalisation of refrigeration as a way to conserve food, thereby reducing the consumption of food items with a high content in salt and nitrates.

Colorectal cancer mortality is relatively constant for males and decreases slightly for females. Ongoing prevention studies may lead to more precise identification of suspected aetiologic factors, either protective (vitamin A, a diet with a high fibre content, etc.) or detrimental (fat).

The increase in breast cancer mortality is very important for women aged 35-64. In 1985, breast cancer caused about three times more deaths than colorectal cancer which is the second most common cause of death by cancer in females. Breast cancer is now almost as frequent in France as in the USA, which is very different from the situation in the 1950s.

The decrease observed for cancer of the uterus is probably due to a decrease of mortality from cancer of the cervix

**Figure 4** Mortality trends for tobacco and/or alcohol-related cancer sites, French males, age 35-64, logarithmic scale.

**Figure 5** Mortality trends for tobacco and/or alcohol-related cancer sites, French females, age 35-64, logarithmic scale.

rather than from the uterine corpus. This decrease is probably related both to improvements in hygiene and to screening leading to treatment of precancerous lesions and earlier treatment of cancers.

The general trends in cancer mortality observed in France are quite similar to what is observed in other Western countries: for males, one observes an overall increase, mostly due to respiratory and upper digestive tract cancers, and a decrease for stomach cancer; for females, one observes an overall moderate decrease, mostly due to cervix and stomach cancer, and an increase in breast cancer mortality. The particularities of cancer mortality in France compared to other countries are the low lung cancer mortality rate in females and the high mortality for mouth, pharynx and oesophagus.

### References

HILL, C., BENHAMOU, E., DOYON, F. & FLAMANT, R. (1989). *Evolution de la mortalité par cancer en France entre 1950 et 1985*. INSERM: Paris.

HIRSCH, A., HILL, C., FROSSART, M., TASSIN, J.P. & PECHABRIER, M. (1987). *Lutter contre le tabagisme*. La documentation française: Paris.

LION, L., HATTON, F., MAGUIN, P. & MAUJOL, L. (1988). *Statistiques médicales des causes de décès 1985*. INSERM: Paris.

PERCY, C.L. (1981). Conversion of neoplasm section, 8th revision of international classification of diseases (1965) to 9th revision of international classification of diseases (1975). USDHSS: Bethesda, Md.

WATERHOUSE, J., MUIR, C., CORREA, J. & POWELL, J. (eds) (1976). *Cancer Incidence in Five Continents, Vol III*. IARC: Lyon.